# Splenic Enlargement in Infancy

**Published:** 1909-02-13

**Authors:** 


### DISEASES OF CHILDREN.

### SPLENIC ENLARGEMENT IN INFANCY.

MANY problems of interest in connection with the enlargement of the spleen in infancy await solution. Until we arrive at a more clear and definite knowledge of the etiology and pathology of the hyperplasia, we are seriously handicapped in our methods of treatment and still more in giving a reliable prognosis of the course of events. It is impossible to discuss in a short article all the varieties of splenic enlargement; but it may be worth while to draw attention to the treatment and the outlook in that variety which is recognised under various names, such as von Jaksch's disease, *anæmia splenica infantum* and *pseudo-leukæmia infantum*.

This definite clinical entity is characterised by anæmia and enlarged spleen, without any clear evidence of a primary cause, and with no special changes in the blood corpuscles beyond a certain polymorphous character. It affects both sexes equally, and is most common between six months and two years of age. Many of these infants are rachitic or suffer with intestinal troubles. The liver and lymph nodes may be enlarged. In late stages there may be hæmorrhages, jaundice, and œdema. Pyrexia is not infrequent, and generally due to some complication. The cause is probably a toxin of intestinal origin, giving rise to anæmia and splenic enlargement simultaneously, the hyperplasia not being due to the anæmia, nor the anæmia a secondary effect of the hyperplasia. In fact, most profound anæmia may occur in infants without any evidence of splenic hyperplasia. Syphilis can be excluded as a cause in many of these cases, though it is often found in the history of an anæmic cachectic child with an enlarged spleen. Jews seem more liable than Gentiles. This is a particularly noticeable feature, for the affection is most common in the bottle-fed, especially in those brought up on condensed milk and proprietary foods. Yet the proportion of bottle-fed infants among the Jews is smaller than among the Gentiles.

An illustrative case came under the writer's care a few months ago. It was that of a boy, aged eight months, who was sent for consultation on account of supposed scurvy. He was the first child, born at full time, weighing about eight pounds. He had been entirely bottle-fed on cow's milk for a month, on Clay Paget's modified cow's milk for six months, and since then on cow's milk and Allenbury No. 3 food, condensed milk and Allenbury food, Benger's food and condensed milk. He cut his first teeth at six months, at which time he weighed about fourteen pounds. Apparently the diet had been fairly satisfactory and the progress reasonably good up to this date. He then apparently failed in health, possibly from the too prolonged diet of cooked food, and for the last two months had been ailing, though he was not taken to a doctor until the day before he was sent up to town for consultation.

His doctor reported anæmia, spongy and hæmorrhagic gums, and petechial spots about the body. There was some vomiting and irritability of the bowels. In the evening he had a feed of cow's milk, with the result that the stools became green and contained curds. Examination showed a sallow, anæmic child, with fair general nutrition, but very flabby. He had six teeth, and the gums surrounding the four upper incisors were markedly dusky. The spleen was enlarged and extended an inch below the ribs. There was neither pain nor tenderness on examination, and on these grounds it appeared justifiable to exclude the diagnosis of scurvy; for it is extremely rare to find scurvy present in a sufficiently marked form to cause dusky gums and petechiæ without very distinct and even severe tenderness and pain on movement. Moreover, splenic hyperplasia is not a characteristic of scurvy.

In view of the gastro-intestinal condition the child was ordered condensed milk, 1 in 9 parts of water, for four feeds, and two feeds of Benger's food made with equal parts of milk and water. In addition he was given the juice from raw meat, and a teaspoonful of fruit juice four times a day. For a week he progressed favourably except for an evening rise of temperature to 104° F. one night. Then he lost his appetite, and when seen a fortnight after the first visit he was distinctly more anæmic. He was cutting the lower lateral incisor teeth, and the gums were much less dusky. The spleen had increased considerably in size, and reached the level of the navel. The liver was also enlarged. The treatment was continued, yolk of egg added to the diet, and liquor arsenicalis prescribed.

As might be supposed, the prognosis in such a case was naturally guarded on the first interview and somewhat gloomy on the second. The fact that there had been so little improvement in the general symptoms during the fortnight and a distinct increase in the size of the spleen and the liver suggested that the child was rapidly going downhill. Still, part of the evil might have been due to anorexia from teething, and experience has shown that none of these cases is absolutely hopeless.

Even though the blood changes may be profound recovery is still possible, as in the case of a boy aged 13 months in whose blood a large number of myelocytes were found. In hospital practice the majority of these patients are discharged much improved, but what becomes of them afterwards is uncertain. Some undoubtedly recover, and the spleen reverts to its normal size.

For three months the child remained practically stationary, although he took glycerine extract of bone marrow all the time, and arsenic for the first two months. He then was ordered a change of air and given a diet of three feeds of Benger's food and two of nursery rusks with milk. After that he steadily and rapidly improved, and at one year of age weighed 22 pounds, had 11 teeth, and seemed perfectly well. Why the sudden improvement took place is not quite obvious. It might be put down to change of air, improved appetite, and better digestion.

The treatment of these cases resolves itself into very careful feeding on a diet containing the fresh elements of food, as far as possible, or amplified by the addition of fresh meat juice, yolk of egg, and fruit juice. Arsenic certainly does good, and possibly bone marrow preparations may be of value. It is important to attend to the state of the stomach and intestines, but I have not found intestinal antiseptics of much use. All other suitable hygienic measures must be insisted upon.

The prognosis is bad in those infants who are profoundly wasted, with œdema of the feet, intestinal catarrh, and hæmorrhages from nose, gums, mucous membranes, and into the skin. Death often results from bronchitis, broncho-pneumonia, intestinal affections, or asthenia.

